# Optokinetic nystagmus reflects perceptual directions in the onset binocular rivalry in Parkinson’s disease

**DOI:** 10.1371/journal.pone.0173707

**Published:** 2017-03-13

**Authors:** Mana Fujiwara, Catherine Ding, Lisandro Kaunitz, Julie C. Stout, Dominic Thyagarajan, Naotsugu Tsuchiya

**Affiliations:** 1 School of Psychological Sciences, Faculty of Medicine, Nursing and Health Sciences, Monash University, Melbourne, Victoria, Australia; 2 Araya Brain Imaging, Tokyo, Japan; 3 Department of Neurosciences, Southern Clinical School, Monash Health, Melbourne, Victoria, Australia; 4 Research Center for Advanced Science and Technology (RCAST), The University of Tokyo, Tokyo, Japan; 5 Monash Institute of Cognitive and Clinical Neurosciences, Monash University, Melbourne, Victoria, Australia; Stanford University School of Medicine, UNITED STATES

## Abstract

Optokinetic nystagmus (OKN), the reflexive eye movements evoked by a moving field, has recently gained interest among researchers as a useful tool to assess conscious perception. When conscious perception and stimulus are dissociated, such as in binocular rivalry—when dissimilar images are simultaneously presented to each eye and perception alternates between the two images over time—OKN correlates with perception rather than with the physical direction of the moving field. While this relationship is well established in healthy subjects, it is yet unclear whether it also generalizes to clinical populations, for example, patients with Parkinson’s disease. Parkinson’s disease is a motor disorder, causing tremor, slow movements and rigidity. It may also be associated with oculomotor deficits, such as impaired saccades and smooth pursuit eye movements. Here, we employed short-duration, onset binocular rivalry (2 s trial of stimulus presentation followed by 1 s inter-trial interval) with moving grating stimuli to assess OKN in Parkinson’s disease patients (N = 39) and controls (N = 29) of a similar age. Each trial was either non-rivalrous (same stimuli presented to both eyes) or rivalrous, as in binocular rivalry. We analyzed OKN to discriminate direction of stimulus and perception on a trial-by-trial basis. Although the speed of slow-phase OKN was slower in the patients, discriminability of conscious perception based on OKN was comparable between the groups. Treatment with anti-Parkinson drugs and deep brain stimulation improved motor ability of patients, but did not impact on OKN. Furthermore, OKN-based measures were robust and their latencies were shorter than manual button-based measures in both groups and stimulus conditions. To our knowledge, our study is the first to demonstrate that OKN can be used as a reliable indicator of conscious perception in binocular rivalry even in Parkinson’s disease patients in whom impaired manual dexterity may render button-press reports less reliable.

## Introduction

By the time motor deficits are apparent in Parkinson’s disease (PD) patients, more than 70% of dopamine-generating neurons are lost [1]. As the neurodegeneration progresses, so does motor disability, manifesting in slowed and small movements (bradykinesia/hypokinesia) and muscle rigidity. Neurodegeneration also affects saccades [2–8], smooth pursuit eye movements [2–6,8,9] and visual perception [10]. Reduced mobility creates difficulties for perceptual research on PD patients because impaired motor dexterity renders manual responses unreliable.

Recently, optokinetic nystagmus (OKN), a reflexive eye-movement evoked by a moving field, has been gaining traction among researchers as a tool to assess perception, because it can reliably signal conscious perception under certain experimental paradigms [11,12]. In particular, under binocular rivalry, where dissimilar images are presented to each eye and subjects continuously report changes in their perception from one image to the other, a tight correlation between OKN and perceptual switches has been established primarily in healthy adult humans and macaque monkeys [11,13–19]. However, it remains largely unknown whether such a correlation exists across all populations, including those with brain diseases. The question is of considerable scientific and clinical importance if OKN is to be used as convenient ‘readout’ of perception in clinical populations, such as people who have impaired motor responses due to PD.

To use the OKN as a “readout” of perception in PD, the OKN of patients must be intact, and must reflect perception. However, the effect of PD on the OKN is controversial; some studies reporting impaired horizontal OKN in PD patients [6,7,9] while others claim it is intact [3–5,8,20,21]. Moreover, there are no studies on the OKN and perception in PD patients during binocular rivalry. Here, we used the binocular rivalry paradigm to investigate OKN as a “readout” of perception in PD patients. We compared OKN and button press reports in a short-duration ‘onset rivalry’ paradigm [22]. We chose 2 sec for a stimulus duration, rather than a much shorter duration, to reduce the frequency of “fused” percept [23] and to obtain reliable OKN. In this paradigm, rivalrous trials (in which stimuli travelling in opposite directions are projected to each eye) are intermixed with non-rivalrous trials (in which stimuli travelling in the same direction is projected to both eyes). Non-rivalrous trials provide a baseline estimate of the speed and accuracy of OKN and button press. We hypothesized that manual and ocular responses would be slowed down in PD patients compared to controls (between-subjects comparisons) as well as within patients when they are off-treatment than on-treatment (within-subject comparisons). Our study design allows us to quantitatively compare the accuracy and latency of manual and ocular responses in rivalrous condition compared against control non-rivalrous condition.

## Materials and methods

### Subjects

Thirty-nine PD patients and 29 age-similar normal controls participated in this study. We tested a further 9 PD patients, but did not include them because they did not complete two testing sessions. Seventeen patients had been implanted with electrodes for deep brain stimulation (DBS) treatment and the other 22 patients were treated only with medication. All PD patients were tested twice—with and without medication or with and without DBS, in counterbalanced order. In the on-medication state, patients took their usual anti-Parkinson medications, whereas in the off-medication state, patients did not take the medication at least 12 hours before the testing. In the on-DBS state, patients completed testing on their usual DBS settings, whereas in the DBS-off state, testing commenced 30 min after DBS was turned off. We did not record the exact time when the medication or DBS was taken off. In each session, the severity of each patient’s motor deficits was measured using the Movement Disorder Society Unified Parkinson's Disease Rating Scale (MDS-UPDRS) Part III (motor examination). All subjects gave written informed consent prior to their participation in the study. The study conformed to the Declaration of Helsinki and was approved by the Monash Health Research Ethics Committee (MUHREC 12350B).

### Apparatus

Stimuli were created using Matlab Psychtoolbox [24,25] with Matlab 2013 on a MacBook Pro and displayed on a 23” Tobii TX-300 screen (resolution: 1920 x 1080 pixels, refresh rate 60 Hz). Eye movements were recorded using a Tobii TX-300 eye tracker (Tobii Technology, Danderyd, Sweden) at a sampling rate of 300Hz. Tobii was controlled by Matlab with the software package T2T (http://psy.cns.sissa.it/t2t/About_T2T.html).

Subjects sat in a brightly-lit room on a height-adjustable chair, with their heads stabilized by a chin rest at a distance of 74 cm from the monitor. Each grating stimulus was projected to each eye, through a custom-built stereoscope consisting of four mirrors. Two of the mirrors were transparent to infrared light, allowing the eye-tracker to track eye position whilst restricting each eye to viewing only one grating [18]. Subjects responded with both hands using numeric keypads. The button press data was recorded in Matlab at the sampling rate of 60Hz.

### Stimuli

We tested subjects’ OKN with moving sinusoidal gratings. The gratings were presented at the center of each mirror, confined within a square area of 5.34 degrees of visual angle. The gratings had a spatial frequency of 0.27 cycles per degrees of visual angle and a temporal frequency of 6.02 cycles per second (i.e., at a speed of 22.3 degrees of visual angle per second). Each grating was framed by a square box of a random texture pattern, which facilitated binocular fusion in subjects.

### Experimental procedures

**Fig 1:**
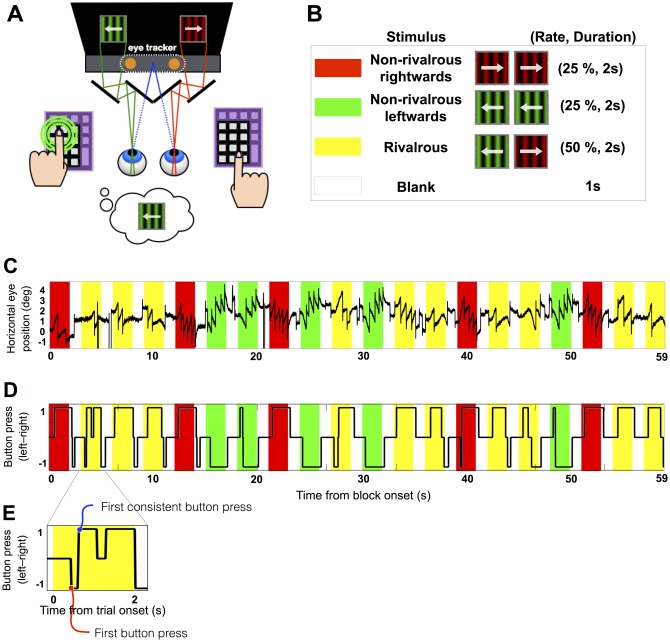
Experimental paradigm. (A) A mirror stereoscope was used to present distinct images to each eye. The mirrors in front of the subject’s eyes were transparent to infrared light, allowing for eye tracking. Subjects were instructed to press a key according to their dominant perception: on the left (or right) keypad when the green (or red) gratings appeared to go leftwards (or rightwards). (B) Each block contained 20 repetitions of a 2-s trial with a 1-s blank interval between trials. Non-rivalrous leftwards, rightwards or rivalrous trials were randomly intermixed, composing 25%, 25% and 50% of trials in each block, depicted as red, green and yellow in panel B, C, and D, respectively. (C) An exemplar time course of horizontal eye position in one PD patient for 1 block. Center of the stimulus corresponds to the eye position of 0 deg. In non-rivalrous trials (red and green) there is clear OKN with slow phase moving in the same direction as the stimulus, and fast phase (saccades) moving the eyes back. In this block, horizontal eye position was positively biased towards right. Slight bias is expected as we did not provide a fixation point, which reduces OKN. (D) The button press time course from the same block as above. (E) Magnified button press time course shown in (D). Here, the subject first briefly pressed a left button within the first 0.5 s and then switched to a right button, which they held down for about 0.2 s. Just after 1 s, they briefly let go of the right key before pressing a right key again, until they changed to a left key just before the end of the 2 s trial. In this case, the most consistently endorsed direction (and thus the labelled direction of this trial) is “right”, the first button press time is 0.5 s and the and first consistent button press time is 0.6 s.

Subjects viewed stimuli through a mirror stereoscope (Fig 1A). Before starting the experiments we checked that they had achieved binocular fusion. Subjects were instructed to report the dominant direction of motion of the gratings as leftwards (or rightwards) with their left (or right) hand, using the keypads (Fig 1A). They were asked to press and hold-down the key as soon as they saw the grating moving left or right. During the 1-s inter-trial interval, they were asked to release the button.

Our paradigm utilized a task design with relatively short stimulus duration (2 s), called “onset rivalry” [22], which may have distinct neural mechanisms compared to those that govern continuous stimulation of rivalrous stimuli, which are the target of the majority of rivalry studies [26–29]. We used this paradigm to measure latency from an objective, standardized fixed point (stimulus onset). In contrast, continuous rivalry offers no objective, fixed point from which to measure the latencies of button press and OKN. We chose 2-sec for a stimulus duration to reduce the frequency of “fusion” percept [23], which tends to increase with shorter duration onset rivalry.

Before the main experiment, subjects practiced the task for 4 blocks of 30 s. During practice, we confirmed: 1) that only one button was pressed not both at the same time, 2) that button presses were accurate in non-rivalrous trials, and, 3) that the button was released during the blank periods. We confirmed these online by the time course plot (similar to Fig 1D).

Before each block of the main experiment, we checked binocular fusion and calibrated the eye-tracker using our custom-written 5-point calibration program that accommodates the mirror setup. After each block, we re-calibrated the eye-tracker if less than 80% of data was validly recorded according to Tobii’s criteria. Eye movements were recorded for each block of 60 s, which contained 10 non-rivalrous and 10 rivalrous trials, randomly intermixed (Fig 1B, 1C and 1D). An experimental session consisted of 8 blocks (~40 minutes; due to a technical error, we failed to record the timing signal in the last block of each session, leaving first 7 blocks for the analysis). Subjects were allowed to take rests anytime in between blocks if they felt tired or drowsy. When time allowed, subjects were tested with further blocks if recording of eye gaze data was poor in the preceding blocks.

### Behavioral analysis

From the button press data, we obtained standard summary statistics (e.g., latency and accuracy). Due to the nature of the task design, the definitions of these concepts are rather involved as detailed below.

Before proceeding with eye tracking analyses, we excluded trials and subjects based on the button press data (Table 1). First, we excluded trials if the button was not released during the inter-trial interval, as we could not define the button latency in such a trial. We also excluded trials with no or double button presses for more than 1 s during the 2 s of trial. Second, we rejected subjects if more than half of their rivalrous or non-rivalrous trials were rejected.

**Table 1:** Rejection of trials and subjects. For details, see Method. Subjects were rejected if more than half of trials were rejected. Note that controls were tested in one session while PD were tested two sessions, explaining a larger number, but comparable ratio, of rejected trials. “Effect of both” represents effect of both button press and eye movement. “Imbalance” represents imbalance of perceptual direction, meaning that less than 3 trials were classifiable as either dominantly- left or right responses.

	Subject Group	Control	PD
	Tested subjects	29	39
Button Press	Rejected trials due to BP	1363 (29.1%)	4290 (33.1%)
	Rejected subjects	4 (13.8%)	9 (23.1%)
	Remaining subjects after rejection due to BP	25	30
Eye Movement	Rejected trials due to eye movement	1139 (24.3%)	3538 (23.9%)
	Rejected subjects	0	2 (7.7%)
Effect of both	Rejected trials in total	1685 (35.4%)	5204 (40.1%)
	Rejected subjects	1 (4.0%)	0
	Remaining subjects after rejection due to eye movement	24	28
Imbalance	Rejected subjects	0	6 (15.4%)
Total	Final rejected subjects	5 (17.2%)	17 (43.6%)
	Final subjects	24	22

In non-rivalrous condition, accurate button press is objectively defined. To calculate button response accuracy, the direction of the stimulus in each trial (StimDir) was assigned -1 for left and +1 for right. The state of button press at time = t (RawBP(t)), was assigned -1 for left, +1 for right, and 0 for no or double button press. Then, we smoothed this RawBP with a 100 ms boxcar kernel to obtain the button press at time t: BP(t). Next, we defined correctness of button press at time = t as C(t), which is 1 if BP(t) * StimDir > = 0.5 and 0 otherwise.

In rivalrous condition, the accuracy cannot be objectively defined. Instead, we labeled each trial either as dominantly- left or right, based on dominant button press in that trial. Then we examined the relationship between the dominant percept of a trial and the button press at a given time of the trial. For this purpose, first we calculated the dominant button press for a given trial (pBP) by averaging BP(t) from t = 0 s to t = 1.5 s. Then, we labeled the trial as dominantly-left (LabelDir = -1) if pBP <0 and dominantly-right (LabelDir = 1) if pBP >0. Then, we defined button press consistency C(t), which is 1 if BP(t) * LabelDir > = 0.5 and 0 otherwise. (In the non-rivalrous trials, consistency of button press ends up almost identical to button press correctness because >92% of trials were correct and consistent. Thus, we intentionally use the same abbreviation C(t) to compare non-rivalrous and rivalrous trials.) Button response accuracy for a given trial was defined as as the mean C(t), where t ranges from the first button press time at each trial to 1.5 s from the stimulus onset.

As seen in Fig 1E, subjects often switched the button press, which may reflect erroneous response or genuine perceptual switch. To investigate a possible speed-accuracy tradeoff, we analyzed “first button press time” and “first consistent button press time” (Fig 1E). ‘First button press’ is the absolute first button pressed in each trial and disregards subsequent button press switches, including any corrective switches if the first button press was made in error. ‘First consistent button press’ is the first button press consistent with the labelled direction of that trial (i.e. calculated as when BP(t) * LabelDir > = 0.5) (for an example, see Fig 1E).

### Eye movement data analysis

From the eye movement data, we obtained mean slow-phase OKN velocity and classification accuracy of percepts based on this signal.

The velocity of slow-phase OKN has been shown to provide a continuous and stable estimate of conscious perception [13–18]. We performed the following preprocess to obtain the velocity of slow-phase OKN.

First, we removed blinks (i.e., periods of missing fixation position data) and saccades, which we identified with a threshold of 6 deg/s in velocity and 1 deg/s^2^ in acceleration [30]. Denoting the i-th removed time period as [R_start_(i), R_end_(i)] and x-coordinate of the fixation position at time = t as F(t), we interpolated F(t) over the time period t = [R_start_(i) - 10 ms, R_end_(i) + 10 ms] with a constant value of F(R_start_(i) - 10 ms). After interpolation, the subsequent fixation position (i.e., F(t) where t > = R_end_(i) + 10 ms) was shifted so that it started from the interpolated position, that is, F(t) was replaced with F(t)—F(R_end_(i) + 10 ms) + F(R_start_(i) - 10 ms) for t > = R_end_(i) + 10 ms. We repeated this procedure until we removed all blinks and saccades to obtain concatenated fixation data which we call ‘integrated OKN’.

Second, we smoothed the integrated OKN with the same boxcar kernel of 100 ms that we applied to button press time course. We then computed instantaneous velocity of integrated OKN as the difference between neighboring two time points (3.3 ms difference). To obtain a velocity of the slow-phase OKN, we further smoothed the instantaneous velocity with the 100 ms boxcar kernel. Finally, we segmented the time course of the velocity of slow-phase OKN from 1 s before to 2 s after the onset of stimuli. We did not include the first trial of each block in the analysis as we did not record fixation position before the first trial.

We rejected trials if the total length of blinks, saccades and undetected time points by the eye tracker within the 2 s trial exceeded 1 s. If we rejected more than half of trials due to either button press criteria (mentioned above) or eye tracking criteria, we excluded the subject. After these rejection, we also rejected subjects who had less than 3 trials classifiable as either dominantly- left or right responses (See Table 1 for details).

### Support vector machine classification on button press and OKN

To quantify discriminability of perceptual report in OKN and button press, we employed a supervised classification algorithm, called support vector machine (SVM) with a single feature: either an instantaneous velocity of slow-phase OKN or BP at time = t.

To avoid a bias introduced by an unequal number of trials, we first subsampled the same number of the trials (N). N is the smaller of the number of right (NR) and left (NL) trials (i.e., N = min [NR, NL]). For training, we used 70% of the subsampled trials (N). For testing, we used the weight from the trained SVM to classify the remaining 30% of the trials, called a test set. We obtained cross validation accuracy as the mean accuracy of the classifier on the test set. We computed 10 cross validation accuracy by repeating the procedure from subsampling and regarded the mean of the 10 cross validation accuracy as discriminability. We obtained discriminability at each time point, each condition and each subject independently.

## Results

**Fig 2:**
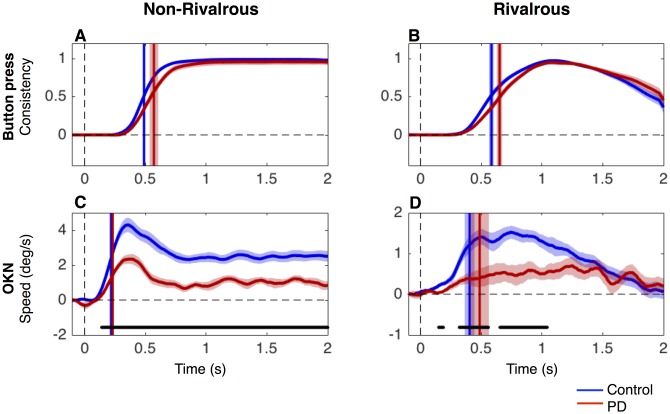
Time course of button press consistency and OKN. The mean time course of button press consistency (A and B) and velocity of slow-phase OKN (C and D) in non-rivalrous (A and C) and rivalrous (B and D) conditions. The vertical lines denote the latency, defined as the time to reach half of the maximum. Black lines at the bottom (only present for C and D) represent the period of statistical difference between PD and controls (unpaired two-tailed t-test, False Discovery Rate adjusted at q = 0.05, p<0.05). The data for PD patients and controls are shown in red and blue, respectively, and the shaded area (when visible) represents SEM across subjects. Note that y-axis for C and D are on different scales.

**Fig 3:**
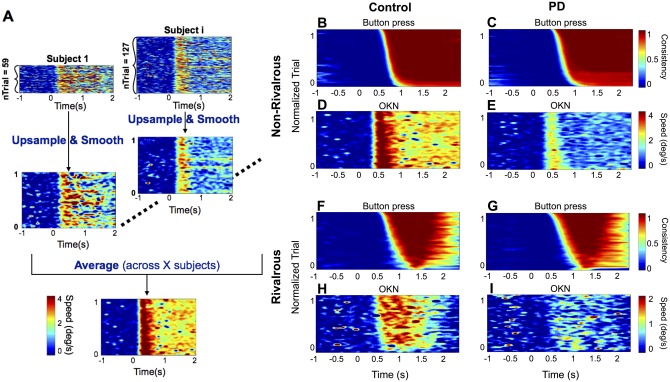
Trial-by-trial image analyses for button press and OKN. A) Preprocessing for image analyses. We first sorted the order of trials according to the button-press consistency, C(t) (see Methods), over each 2 s trial. Next, we stretched the trial x time image along the trial dimension so that we can average the image across subjects who have different numbers of valid trials. Then, we upsampled the image by 1000 points along the trial dimension. Next, we smoothed the image by a boxcar kernel of 31 points along the trial dimension and averaged across subjects. This process was performed for both button press consistency and OKN. B-I) The normalized trial (stretched and upsampled) x time images for button-press consistency (B, C, F and G) and OKN (D, E, H and I), across controls (B, D, F, H) and PD patients (C, E, G, I). Note that button press and OKN velocity was flipped for the left trials (as was done for Fig 2). Button press consistency and OKN speed are denoted according to the color scale key on the right.

**Fig 4:**
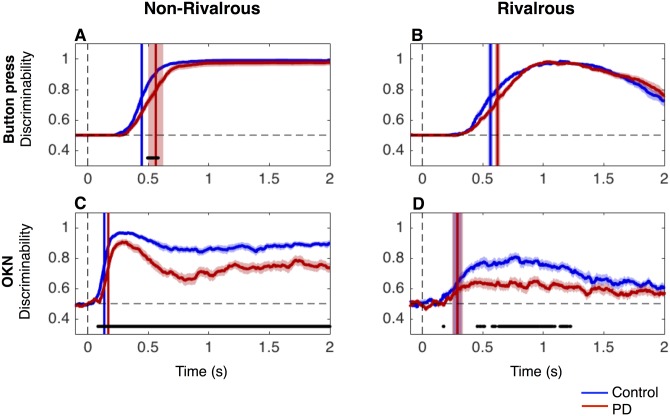
Discriminability analyses. The mean time course of the discriminability of the direction of physical stimuli in non-rivalrous condition (A and C) or dominant percept in rivalrous condition (B and D), measured with button press consistency (A and B) and OKN (C and D). Red and blue lines for PD patients and controls, respectively. The vertical lines denote time to reach half of the maximum discriminability. Shaded area (when visible) represents SEM across subjects. Black lines at the bottom indicate time points with significant difference between groups (p<0.05 with FDR q = 0.05).

**Fig 5:**
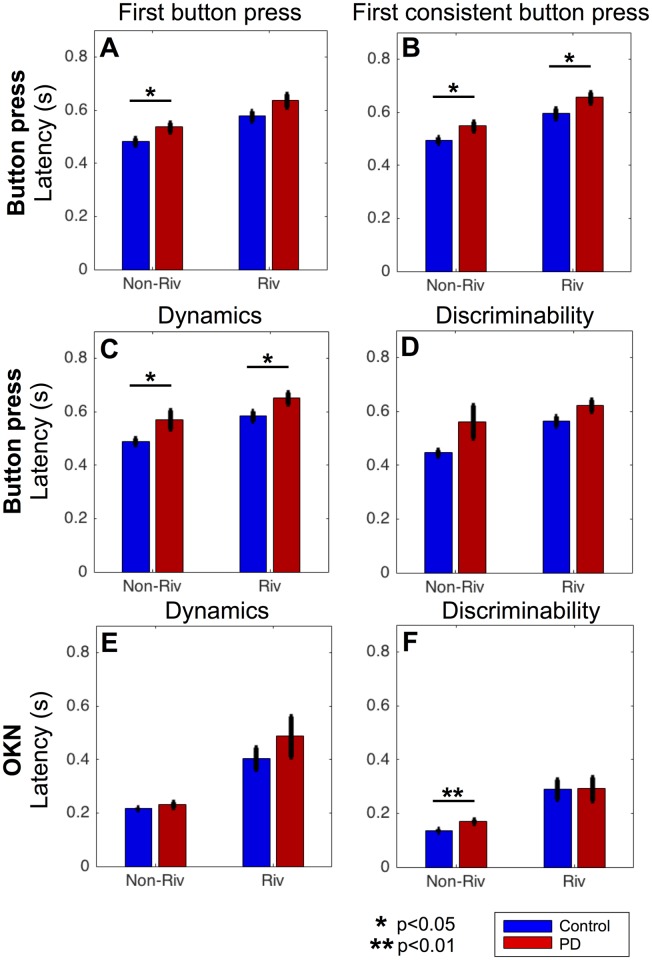
Summary of measures comparing PD (on medication or DBS treatment N = 22) and control (N = 24) in non-rivalrous and rivalrous condition. Results for button press (A-D) and OKN (E and F) for patients with Parkinson’s disease (red) and controls (blue). In each panel, the non-rivalrous (non-riv) condition is on the left and the rivalrous (riv) condition is on the right. Error bars represent SEM across subjects. * and ** indicate significant difference between PD patients and controls at p<0.05 and p<0.01 respectively (unpaired two-tailed t-test).

**Fig 6:**
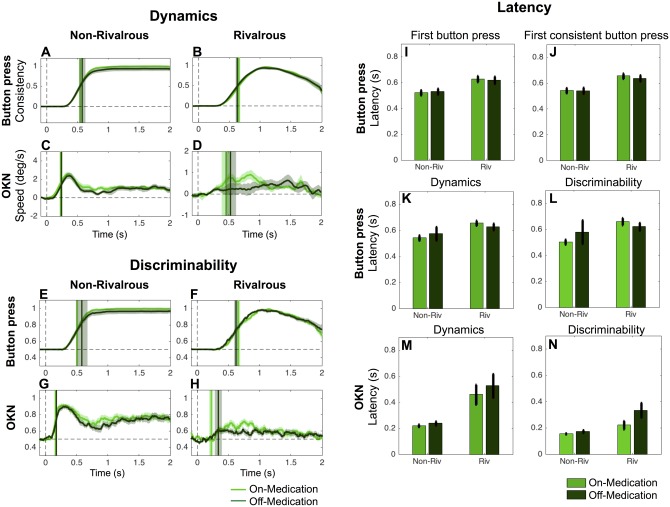
No effects of medication on button press and OKN (within subject comparison). The format of panel A-D, E-H, and I-N is the same as for Figs 2, 4 and 5. N = 15 subjects were tested in the on- (light green) and off- (dark green) medication state. Note that y-axis for C and D are on different scales. No significant differences were found (FDR q = 0.05 for corrections of multiple comparisons across time for A-H).

We studied the effects of PD and its treatment on behavioural responses and OKN in three separate analyses. In the first set of analyses (Figs 2–4), we examined the effects of PD on button press and eye movements in PD patients when they are on treatment (either on-medication or on-DBS), comparing with controls. In the second (Fig 5) and third (Fig 6) set of analyses, we investigated the within-subject effects of the medication and DBS, respectively.

Table 2 summarizes subject demographics for each group in the first set of analysis. Although rejection rate of PD patients was significantly higher than controls (chi^2^ = 5.28, p = 0.022), any particular rejection criteria did not differ significantly between the groups (button press: chi^2^ = 1.17, p = 0.28; eye movements: chi^2^ = 0.65, p = 0.42; both: chi^2^ = 3.56, p = 0.6; imbalance: chi^2^ = 2.43, p = 0.12) (Table 1).

**Table 2:** Demographics for subjects included for the first set of analysis, which compared controls and PD patients in on-treatment session. MDS-UPDRS score for PD patients are the mean of 21 patients, because we could not obtain MDP-UPDRS in one PD patient for one of their two sessions (the on-DBS session).

Subject Group	Control	PD On-treatment
Analyzed subjects	24	22
Age	65.0 (7.7 SD)	66.5 (8.0 SD)
MDS-UPDRS	NA	13.6 (7.6%)

### PD patients can report their percepts with buttons as accurately as, but more slowly than controls

First, we examined the time of button press for perceived direction of motion in non-rivalrous conditions. PD patients reported the correct direction as accurately as controls (patients: M = 92.5%, SEM = 0.93; controls: M = 93.6%, SEM = 0.55; unpaired two-tailed t-test: t(44) = 0.98, p = 0.32). However, reaction times, measured as the first button press times, were prolonged in patients (M = 536 ms, SEM = 16.7) compared to controls (M = 482 ms, SEM = 13.3; unpaired two-tailed t-test: t(44) = -2.55, p = 0.014).

Next, we examined the time of button press in rivalrous condition. While we cannot define the accuracy for rivalrous condition as for non-rivalrous condition, we can compare two types of button press latency: the latency to first button press and the latency to first ‘consistent’ button press (as defined in Methods. See Fig 1E). The mean latency of first button press in rivalrous condition did not differ between the groups (patients: M = 636 ms, SEM = 24.8; controls: M = 579 ms, SEM = 18.8; unpaired two-tailed t-test: t(44) = -1.85, p = 0.071). The latency of first ‘consistent’ button press takes into account of both potential perceptual switch during the 2 s trial and an initial impulsive (and brief) button press followed by a second corrective (and more prolonged) button press (see Method). The mean latency of first consistent button press in rivalrous condition was significantly slower for patients (M = 655 ms, SEM = 21.6) than for controls (M = 596 ms, SEM = 18.0; unpaired two-tailed t-test: t(44) = -2.13, p = 0.039).

Taken together, we conclude that PD patients can respond as accurately as, but more slowly, than controls with button press in non-rivalrous condition. In rivalrous condition, patients showed slower consistent responses than controls.

### Temporal dynamics of button press reports and OKN

Next, we analyzed the mean time course of the button press and the velocity of slow-phase OKN (Fig 2).

For button press, we analyzed whether it was consistent with the labelled direction of the trial over time. Fig 2A and 2B confirm that button press in PD patients is slower than in controls.

Although the mean button press consistency in patients appears lower than that of controls between 0.4–0.8 s for both conditions (Fig 2A and 2B), the differences did not reach significance (unpaired two-tailed t-test at each time point, multiple comparisons were corrected with False Discovery Rate at q = 0.05). Due to the smoothing procedure, we expected that the latency for the first consistent button press would roughly correspond to when consistency reached 0.5 As expected, the time to reach 0.5 in button press consistency was significantly slower in PD patients (Fig 2A and 2B, vertical lines) than controls for both non-rivalrous (unpaired two-tailed t-test: t(44) = -2.24, p = 0.030) and rivalrous condition (unpaired two-tailed t-test: t(44) = -2.23, p = 0.030).

Fig 2C and 2D show the mean time course of the velocity of slow-phase OKN. Note that we flipped the sign of the OKN for the left trials which tend to have negative velocity and that we scaled the y-axis differently for Fig 2C and 2D. Patients had a slower OKN than controls in both non-rivalrous (about 1.5 dg/s slower in patients, p<0.05 from 0.15 s to 2.0 s) and rivalrous conditions (about 1 dg/s slower in patients, p<0.05 from 0.15 s to 1.1 s indicated by black lines at the bottom of Fig 2D; unpaired two-tailed t-test at each time point, with FDR q = 0.05).

The latencies of OKN, defined as the time taken for OKN speed to reach the half of the maximum (Fig 2C and 2D vertical lines), were much faster than the button press latencies (Fig 2A and 2B vertical lines). Three-way ANOVA of latency confirmed that significant main effects for the measure (button press vs OKN; F = 97.00, p<0.001), the stimulus condition (rivalrous vs non-rivalrous; F = 40.80, p<0.001) and the subject group (PD patients vs controls; F = 6.33, p<0.05). As can be seen from Fig 2, OKN latency is much faster than button press especially in non-rivalrous condition, which is confirmed by a significant interaction between the measure (button press vs OKN) and the stimulus condition (rivalrous vs non-rivalrous) (F = 7.6, p<0.01). Other interactions were not significant (stimulus*subject group: F = 0.32, p = 0.57; subject group*data type: F = 0.27, p = 0.60; stimulus*subject group*data type: F = 0.75, p = 0.39).

While OKN has a relatively short latency, its reliability in discriminating the direction of perceived motion cannot be inferred from the mean time course. Thus, we turn our focus to trial-by-trial analysis to quantify how accurately OKN reflects conscious perception over time in each trial.

### Trial-by-trial analysis of button press and OKN

To quantify how accurately the OKN reflects the (non-rivalrous) stimuli or (rivalrous) percepts, we performed image-based trial-by-trial analysis. Within each subject, we first sorted the trials by the mean button-press consistency value (over each 2-s trial) in descending order, combined them to form a mean button press consistency x time image. We then stretched the image to a uniform height, upsampled and then, smoothed (only along y-dimension, but not across time). Finally, we averaged the images across subjects to obtain the mean button press consistency x time image for each group. (see Fig 3A).

In the mean button-press consistency x time images (Fig 3B, 3C, 3F and 3G), the color of each pixel represents the button press consistency at a particular time averaged across subjects. The upper rows in each panel represent trials with higher button-press consistency, which tend to have a shorter latency to first consistent button press; the lower rows represent trials with lower button-press consistency, which tend to have a longer latency to first consistent button press and have less time remaining to hold down that button before the trial ends. In this format, perceptual switches in rivalrous condition (Fig 4F and 4G) are captured by the lower consistency value towards the end of the trials.

Two important insights about OKN emerge from the image-based trial-by-trial analysis. First, OKN latency appears shorter and more uniform across trials compared to button press in both subject groups and stimulus conditions. This corroborates the observation in Fig 2. Second, OKN speed is quite variable across trials. To quantitatively compare the latency and variability of OKN with respect to those of button press consistency, we turn to the classification analysis, next.

### Classification analysis: Discriminability of direction of stimulus and perceived motion based on the momentary button press and OKN

Taking into consideration OKN’s variable speed but shorter and more uniform latency compared to button press, we employed support vector classification analysis to quantify how reliably the velocity of slow phase OKN discriminates direction of non-rivalrous stimuli or conscious perception of rivalrous stimuli in patients and controls (see Method).

We first computed the ability of button press to discriminate direction of non-rivalrous stimuli using the button press consistency (Fig 4A), which is a continuous measure after smoothing button presses over 0.1 s—the same amount of smoothing applied to OKN. The maximum discriminability based on the button press consistency reached ~98% (mean across subjects) by 0.5 s (where chance performance is 50% and perfect performance is 100%). Patients did not take longer time than controls to reach half maximum discriminability (vertical lines in Fig 4A) (unpaired two-tailed t-tests: t(44) = -1.90, p = 0.062).

In rivalrous condition (Fig 4B), we labelled trials according to the dominant direction of perceived motion according to button press report during the first 1.5 s (see Methods). We computed discriminability of the labelled direction based on button press consistency value at any one moment. The mean discriminability reached 100% by 1 s. As with non-rivalrous condition, the time to reach the half-maxima did not significantly differ between patients and controls (unpaired two-tailed t-tests: t(44) = -1.98, p = 0.054).

Applying the same analysis to OKN in non-rivalrous condition (Fig 4C), we found the maximum discriminability of the OKN measure reached above 90% (mean across subjects) within 0.3 s for both patients and controls. In accord with previous analyses (Figs 2C, 3D and 3E), the speed of OKN decreased and became more variable across trials after 0.4 s, especially in the patient group. OKN-based discriminability was lower in PD patients than in controls from ~100 ms (p<0.05 with FDR q = 0.05, black lines at the bottom of Fig 4C).

In rivalrous condition (Fig 4D), OKN-based discriminability was reduced compared with non-rivalrous condition, which is evident for the mean time course across subjects. The maximum discriminability reached in each subject was also lower for patients (M = 83.7%, SEM = 2.3) than controls (M = 91.6%, SEM = 1.5; unpaired two-tailed t-test: t(44) = 2.93, p = 0.005).

By defining the latency of discrimination as the time to reach the half of maximum discriminability in each subject, we found that, the latency for OKN discriminability was ~0.15 s faster than for button press in both patients (paired two-tailed t-tests: t(21) = 6.31, p<0.0001) and controls (paired two-tailed t-tests: t(23) = 24.8, p<0.0001) in non-rivalrous condition. The results for the rivalrous condition was similar (Fig 4B and 4D): the latency for OKN discriminability was faster than for button press discriminability in both patients (paired two-tailed t-tests: t(21) = 7.38, p<0.0001) and controls (paired two-tailed t-tests: t(23) = 6.95, p<0.0001). We repeated the above analysis by systematically changing the latency criterion of the discriminability from 0.5 (chance) to 1.0 (perfect). The results did not differ from the above analysis, generalizing our conclusion that the discriminability latency for OKN is faster than that for button press (data not shown).

Taken together, we conclude that OKN reflects the direction of the physical stimuli and dominant percept much faster than button press at a comparable accuracy.

### Summary of results

Fig 5 summarizes the results of button press and OKN for each subject group and stimulus condition. OKN (Fig 5E and 5F) has a shorter latency than button press (Fig 5A–5D) across all analysis methods for both subject groups and stimulus conditions.

We expected PD would delay motor responses across all response modalities, from voluntary button press to involuntary oculomotor reflexes. However, it is notable that on OKN-based measures, patients were significantly slower than controls on the OKN-based discriminability measure only in non-rivalrous condition.

Next we examine the effect of anti-Parkinson medication and deep brain stimulation.

### No effects of anti-Parkinson medication on button press and OKN in both non-rivalrous and rivalrous condition

Dopaminergic medication, such as L-DOPA, reduces motor symptoms of PD, including tremor and rigidity. To investigate effects of medication on button press and OKN in non-rivalrous and rivalrous conditions, we examined 15 medically-treated (i.e. non-surgically treated) PD patients (a subset of PD patients included for the analyses so far) in on- and off- medication states. On-medication was defined as taking their usual anti-Parkinson medication, while off-medication was defined as after withdrawal of all anti-Parkinson drugs for at least 12 hours. Of the 22 patients tested on and off medication, 7 were rejected based on the criteria detailed in Methods. Table 3 lists the details of the patients included in this study. MDS-UPDRS motor disability scores were significantly improved by medication (on-medication: M = 13.9, SD = 8.4; off-medication: M = 21.8, SD = 11.4; paired two-tailed t-test: t(14) = -3.59, p<0.01).

**Table 3:** Subject demographics and rejected trials and subjects in the analyses comparing PD patients on and off medication. Trials were rejected due to the criteria for button press and eye movements (Table 1, see Methods). Subjects were rejected if more than half their trials in either of their on or off sessions was rejected. In total, we rejected 7 subjects (4 patients were rejected both sessions while the other 3 patients were rejected in one of the sessions).

	Subject Group	On-Med	Off-Med
	Tested subjects	22	22
Button Press	Rejected trials due to BP	1392 (29.5%)	1195 (30.5%)
	Rejected subjects	2 (9.1%)	2 (9.1%)
	Remaining subjects after rejection due to BP	20	20
Eye movement	Rejected trials due to eye movement	1228 (26.1%)	936 (23.9%)
	Rejected subjects	0	1 (4.6%)
Effect of both	Rejected trials in total	1744 (37.0%)	1405 (35.8%)
	Rejected subjects	1 (4.6%)	0
	Remaining subjects after rejection due to eye movement	19	19
Imbalance	Rejected subjects	3 (13.6%)	2 (9.1%)
Total	Rejected subjects in total	6 (27.3%)	5 (22.7%)
	Remaining subjects	16	17
	Final rejected subjects	7 (31.8%)	
	Final subjects	15	
Age (±SD) of final subjects		70.0 (6.2 SD)	
MDS-UPDRS (±SD) of final subjects		13.9 (8.4 SD)	21.8 (11.4 SD)

For these analyses, we used the same procedure described in the patients vs controls comparison (Fig 5) to compare patients on and off medication, except that we used paired t-tests in place of unpaired t-tests. As is clear from Fig 6, we found no effect of medication on either button press or OKN, despite significant effects in MDS-UPDRS motor disability scores (on medication: M = 13.9, SD = 8.4; off medication: M = 21.8, SD = 11.4; paired two-tailed t-test: t(14) = -3.59, p = 0.003).

### DBS facilitates button press in rivalrous condition, but did not affect on OKN and button press in non-rivalrous condition

Severe Parkinsonian symptoms, inadequately controlled by medication, can be effectively treated with Deep Brain Stimulation (DBS) [31]. To investigate effects of DBS on button press and OKN in non-rivalrous and rivalrous conditions, we tested 17 patients (a subset of PD patients included in the initial analysis) on and off-DBS states. In the on-DBS condition, DBS settings were the patients’ usual therapeutic settings, while in the DBS-off condition, stimulation was turned off for at least 30 minutes before commencement of testing. Twelve of the DBS patients were also taking anti-Parkinson medication but there was no manipulation of their anti-Parkinson drugs.

Of the 17 PD patients tested on and off-DBS, 10 were rejected, leaving only 7 subjects in the final analysis (see Table 4 for details) Turning off DBS significantly increased UPDRS score for 6 subjects from M = 12.8 (SD = 5.9) to M = 30.2 (SD = 9.7; paired two-tailed t-test: t(5) = -4.15, p = 0.0089. Note that we could not obtain UPDRS in on-DBS session for one patient, leaving us 6 subjects for UPDRS comparison).

**Table 4:** Subject demographics and rejected trials and subjects in the analyses comparing PD patients on and off DBS. Trials were rejected due to the criteria for button press and eye movements (Table 1) that were explained in Methods. Subjects were rejected if more than half of trials in either of their on or off sessions was rejected. In total we rejected 10 subjects; 3 were rejected in both sessions, while the other 7 were rejected in one session (6 in the off-session and 1 in the on-session). MDS-UPDRS score are the mean of 6 patients, because we could not obtain MDP-UPDRS in the on-DBS session.

	Subject Group	On-DBS	On-DBS
	Tested subjects	17	17
Button Press	Rejected trials due to BP	333 (26.9%)	311 (28.8%)
	Rejected subjects	2 (11.8%)	6 (35.3%)
	Remaining subjects after rejection due to BP	15	11
Eye movement	Rejected trials due to eye movement	347 (28.0%)	268 (24.8%)
	Rejected subjects	1 (5.9%)	1 (5.9%)
Effect of both	Rejected trials in total	434 (35.0%)	386 (35.7%)
	Rejected subjects	0	0
	Remaining subjects after rejection due to eye movement	14	10
Imbalance	Rejected subjects	1 (5.9%)	2 (11.8%)
Total	Rejected subjects in total	4 (23.5%)	9 (52.9%)
	Remaining subjects	13	8
	Final rejected subjects	10 (58.8%)	
	Final subjects	7	
Age (±SD) of final subjects		66.5 (8.0 SD)	
MDS-UPDRS (±SD) of final subjects		12.8 (5.9 SD)	30.2 (9.7 SD)

**Fig 7:**
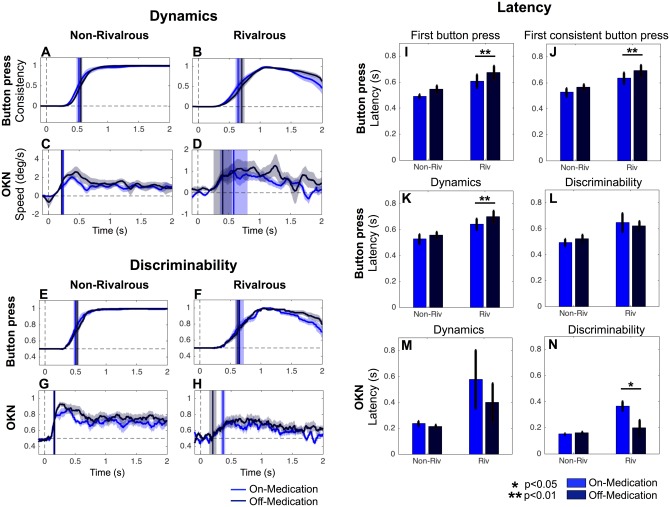
Deep brain stimulation facilitated button press but did not affect OKN. The format of the figure is the same as Fig 6. * and ** indicates significant difference (p<0.05 and p<0.01, respectively). For A-H, corrections of multiple comparisons across time were performed with FDR at q = 0.05.

Fig 7 summarizes the results. We found that DBS reduced the latency of three button press related measures in rivalrous condition; latency to first button press (on-DBS: M = 609 ms, SEM = 51; off-DBS: M = 675 ms, SEM = 51; paired two-tailed t-test: t(6) = -4.20, p<0.01), latency to first consistent button press (on-DBS: M = 637 ms, SEM = 43; off-DBS: M = 697 ms, SEM = 44; paired two-tailed t-test: t(6) = -5.23, p<0.01) and the latency for button press consistency to reach half of the maximum; on-DBS: M = 642 ms, SEM = 41; off-DBS: M = 702 ms, SEM = 44; paired two-tailed t-test: t(44) = -8.29, p<0.01). We observed DBS prolonged latency for OKN speed to reach half of the maximum discriminability in rivalrous condition (on-DBS: M = 366 ms, SEM = 35; off-DBS: M = 199 ms, SEM = 59; paired two-tailed t-test: t(6) = 3.66, p = 0.011).

### No difference between PD treated with medication or DBS

**Fig 8:**
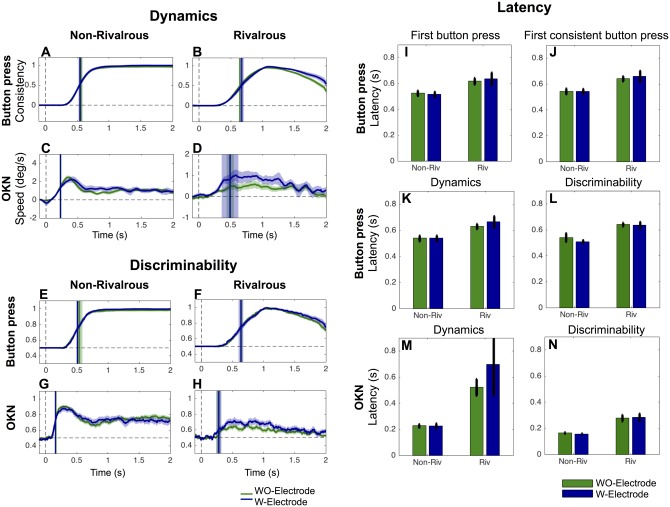
Treatment types of PD did not affect on our measures of button press and OKN. The format of the figure is the same as Figs 6 and 7. The N = 15 medication patients and N = 7 DBS patients were compared after averaging the data for two sessions for each subject. No significant differences were found.

Finally, we examined if patients treated with DBS (N = 7, reported in Fig 7) and medication (N = 15, reported in Fig 6) differed in any aspects of the button press or OKN because baseline severity of disease may have been greater in the DBS group. However, MDS-UPDRS motor score was not different between the groups (unpaired two-tailed t-test; on-state, t(19) = 0.27, p = 0.79; off-state, t(19) = -1.58, p = 0.13). Further, the between groups analysis did not reveal any difference in button press and OKN as summarized in Fig 8 (We averaged the data across on and off states in each subject, then performed the between-subject analysis with unpaired t-tests).

## Discussion

We investigated conscious perception in PD patients using OKN in addition to manual button responses. We found that OKN responses reflected perceptions in PD patients as accurately as in normal controls. OKN proved to be not only a reliable measurement of conscious perception but also faster at detecting perceived direction of motion than manual responses in both patients and controls. Our results are consistent with the study on healthy subjects by Naber and colleagues [18], who used a continuous rivalry paradigm and found that manual responses lagged behind the OKN in signaling perceptual switch. In our study, the speed of OKN was decreased in patients, but the latency of OKN did not differ from controls. In contrast, button press latencies were prolonged in PD patients compared to controls, as expected due to their reduced mobility.

Our findings have implications for psychophysical studies of PD patients. Psychophysical experiments performed on PD patients (e.g., [32]) have often employed verbal report by patients followed by delegated button press report by the experimenter to overcome presumed motor difficulties and other difficulties such as drowsiness [33]. However, delegated response procedures are indirect and preclude measurement of reaction times. Consistent with previous psychophysical studies [34,35], our patients were also slower than controls in 6 out of 8 measures of button press (Fig 5). However, the delay was not so dramatic to the extent that it invalidated the button press reports by the patients altogether.

### DBS facilitates button press response latency in rivalrous condition

Effect of DBS treatment was significant in 3 out of 4 measures related to button press in rivalrous condition (Fig 7). This result is consist with a proposal by Frank and colleagues [36] in which they suggested that DBS interferes with patients in their ability to slow down decision making under high-conflict conditions. It is plausible that perceptual conflict is induced in our rivalrous condition and that DBS specifically facilitates quicker decision in rivalrous condition. Interestingly, Frank and colleagues also reported that no such effect was found with dopaminergic medication. This too, is consistent with our observation of the absence of any effect of medication (Fig 6). While the general patterns of the results are in agreement, the nature of conflicts seem quite different between our perceptual rivalry task and Frank’s cognitive conflict task. Further investigations are necessary to understand if DBS acts on the same neural mechanisms that are responsible for resolving perceptual and cognitive conflicts.

### Effects of PD on OKN

Previous reports on the effect of PD on the OKN are mixed; some studies found that the OKN is impaired in PD patients [6,7,9], while others report that their OKN is intact [2,3,21]. In our study, patients had a slower OKN compared with controls, especially in non-rivalrous condition (Fig 2C). However, each individual’s OKN predicted the direction of stimulus (non-rivalrous condition) or perceptual motion (rivalrous condition) to a comparable degree between PD patients and controls, especially at the trial onset. After 0.4 s, however, the discriminability started to diverge between PD patients and control. It is plausible that perceptual rivalry as well as OKN may be supported by distinct neural mechanisms at the onset phase and subsequent continuous phase [22]. We are currently investigating the effects of PD in a continuous rivalry paradigm.

Neither medication (Fig 6) nor DBS (Fig 7) affected much on the OKN. This suggests that reduction of the OKN speed is a stable deficit and not modifiable by manipulating anti-Parkinson drugs or DBS in the short term.

One potential confound on the reduction of the OKN speed is the difference in fixation accuracy. OKN is known to be weaker for peripheral stimulus [37]. Thus it is possible that slower OKN in PD patients might have been caused by their possibly poorer fixation to the central area of the stimuli. To rule out this potential confound, we computed the average distance between gaze data and the stimulus center in each 2-s trial. We found no difference between PD patients (M = 1.57 deg, SD = 0.60) and controls (M = 1.62 deg, SD = 0.50; unpaired two-tailed t-test: t(44) = 0.79). Thus, the difference of the OKN speed cannot be attributed to the difference in fixation between the patients and controls.

Another possible explanation for the weaker OKN in PD is related to attention, which is known to modulate OKN [38,39]. Further experiments will be required to address what types of attention are impaired and how they affect on OKN speed and discriminability in PD patients.

### Rejections of PD patients due to perceptual imbalance

A relatively high rejection rate of severely affected patients prompts cautious interpretation of some of our results.

Around 30% of trials were rejected for both PD patients and controls (Table 1), mainly because we required subjects to release the button every 2 s after the stimulus period. This required considerable effort from subjects, but it was necessary to properly estimate the latency of button press.

A more interesting pattern of rejection is to do with perceptual imbalance (Table 1), because of which PD patients were more likely to be rejected than controls; no controls and 6 patients were rejected, although the rejection rates were not statistically different (chi-square test, p = 0.12). This implies that PD patients may be more likely to stick with one percept across many rivalrous trials. Reduced perceptual switches (also known as “perceptual freezing” or “perceptual memory”) under the intermittent presentation of ambiguous stimuli has been extensively studied in healthy subjects [40,41]. It would be interesting to test whether PD patients show exaggerated perceptual stabilization. Such a study may provide clues to the critical brain loci responsible for perceptual stabilization, as well as perceptual switches—Einhauser et al. [42] have proposed the locus coeruleus as a potential neural locus for perceptual switches during ambiguous stimulation and that norepinephrine, a precursor of dopamine, may be involved. The locus ceruleus is affected early in PD [43]. Future studies employing OKN readout with no report to study in perceptual stabilization in PD patients may be fruitful in elucidating perceptual consequence of depletion of dopamine.

### OKN as a readout of conscious perception

OKN during binocular rivalry with moving stimuli is shown to correlate well with direction of consciously perceived perception in healthy populations [13–18,44]. Here, we extended the generalizability of OKN readout in rivalry to patients with PD.

The intricacies of the relationship between OKN and conscious perception has not been fully elucidated. In fact, direction of eye movements is not always correlated with conscious perception [45]. When orthogonal horizontal and vertical moving gratings are presented to both eyes separately, the eyes move in the average direction of the two gratings, while subjects only perceive either horizontal or vertical direction of motion, demonstrating dissociation of eye movements and percept [46]. Unlike such a study, we used two gratings moving to the opposite directions, which has been shown to induce strong concordance between eye movements and conscious percept [11,18]. Fully elucidating the conditions that promotes concordance or dissociation between eye movements and conscious percept, combined with neuroimaging, will help facilitate our understanding of the neural mechanisms of consciousness. When OKN are concordant with conscious percept, as in our case, OKN can be used as a reliable readout of conscious perception without requiring button press reports [11,12,14,47]. When OKN dissociates from reported conscious perception, OKN can be used to examine the behavioral and neural mechanisms of the non-conscious visual processing [45,46].

Recently, OKN has received strong attention, in its use for no-report paradigms [12], can be used as a potential readout of conscious percep[12]. No-report paradigms, combined with traditional report-based paradigms, allow researchers to separate the neural activity caused by the act of reporting from the neural activity responsible for generating percept itself. For example, Frässle and colleagues demonstrated that fMRI BOLD signals in the prefrontal cortex during binocular rivalry diminished strikingly under the no-report condition compared with voluntary report conditions. This and other convergent evidence from no-report paradigms [12,48] suggests that the activity in the prefrontal cortex may be more related to the act of reporting, and that the prefrontal cortex may not be a critical neural correlate of consciousness as suggested by studies that only included voluntary perceptual reports [49–52].

Our trial-by-trial analyses (Fig 3) in non-rivalrous condition poses an interesting question. There, in addition to being faster than button press, OKN showed less variability in response latency across trials. With our current methods, we cannot tell which measure (button press or OKN) correlates best with the latency of conscious perception of visual motion. Button press is susceptible to various factors, such as motor preparation and attention, with unknown effects on the latency of conscious perception [53]. It is tempting to suggest that OKN’s faster and less variable response might better reflect the onset of conscious perception than the button response modality, which is currently the dominant measure in psychological studies of consciousness. Broadening the response modalities from traditional button press to continuous measures, such as OKN, may be more effective in capturing complex perceptual dynamics during rivalry [27,54] and in elucidating the gradual nature of consciousness and its neural basis [11].
